# Comparing Seizures Captured by Rapid Response EEG and Conventional EEG Recordings in a Multicenter Clinical Study

**DOI:** 10.3389/fneur.2022.915385

**Published:** 2022-06-29

**Authors:** Deepika Kurup, Kapil Gururangan, Masoom J. Desai, Matthew S. Markert, Dawn S. Eliashiv, Paul M. Vespa, Josef Parvizi

**Affiliations:** ^1^Department of Neurology and Neurological Sciences, Stanford University School of Medicine, Stanford, CA, United States; ^2^Department of Neurology, Icahn School of Medicine at Mount Sinai, New York, NY, United States; ^3^Department of Neurology, University of New Mexico School of Medicine, Albuquerque, NM, United States; ^4^Department of Neurology, David Geffen School of Medicine, University of California, Los Angeles, Los Angeles, CA, United States

**Keywords:** electroencephalography, rapid response EEG, reduced channel montage, seizure detection, non-convulsive status epilepticus (NCSE), highly epileptiform patterns

## Abstract

**Objective:**

A recent multicenter prospective study (DECIDE trial) examined the use of Ceribell Rapid Response EEG (*Rapid-EEG*) in the emergent evaluation and management of critically ill patients suspected to have non-convulsive seizures. We present a detailed, patient-level examination of seizures detected either on initial *Rapid-EEG* or subsequent conventional EEG within 24 h to investigate whether seizures were missed on *Rapid-EEG* due to the exclusion of midline/parasagittal coverage.

**Methods:**

We identified from 164 patients studied in the DECIDE trial those who had seizures detected on *Rapid-EEG* but not conventional EEG (*n* = 6), conventional EEG but not *Rapid-EEG* (*n* = 4), or both *Rapid-EEG* and conventional EEG (*n* = 9). We examined the electrographic characteristics of ictal and interictal findings on both devices, especially their detection in lateral or midline/parasagittal chains, and patient clinical histories to identify contributors toward discordant seizure detection.

**Results:**

Seizures detected on both EEG systems had similar electrographic appearance and laterality. Seizures detected only on conventional EEG (within 24 h following *Rapid-EEG*) were visible in the temporal chains, and external clinical factors (e.g., treatment with anti-seizure medications, sedation, and duration of recordings) explained the delayed presentation of seizures. Patients with seizures detected only by *Rapid-EEG* were treated with anti-seizure medications, and subsequent conventional EEG detected interictal highly epileptiform patterns with similar laterality.

**Conclusions:**

Our case series demonstrates that electrographic data obtained from initial *Rapid-EEG* and subsequent conventional EEG monitoring are largely concordant relative to morphology and laterality. These findings are valuable to inform future investigation of abbreviated EEG systems to optimize management of suspected non-convulsive seizures and status epilepticus. Future, larger studies could further investigate the value of *Rapid-EEG* findings for forecasting and predicting seizures in long-term EEG recordings.

## Introduction

Current guidelines recommend that electroencephalography (EEG) monitoring should be initiated within 1 h when non-convulsive seizures or status epilepticus is suspected (1). However, many hospitals lack the capacity to offer conventional EEG monitoring within this timeframe using the International 10–20 system, which has been the gold standard for recording and displaying EEG data since 1958 (2–5). EEG systems with a reduced number of electrodes have been explored as potential alternatives, however the low sensitivity of hairline and subhairline montages observed in prior studies has led to concerns regarding their utility (6–8).

Rapid Response EEG System (*Rapid-EEG*; Ceribell Inc., Mountain View, CA) was developed to facilitate immediate, real-time EEG acquisition to detect seizures and highly epileptiform patterns (HEP). This device consists of a ten-electrode array arranged circumferentially at the hairline to generate an eight-channel bipolar montage (i.e., lateral channels of the International 10–20 system). This circumferential montage has been shown to provide comparable diagnostic information to the conventional EEG system, despite eliminating midline and parasagittal channels (9–12). A recent multicenter prospective clinical study (Does Use of Rapid Response EEG Impact Clinical Decision Making, DECIDE) demonstrated that *Rapid-EEG* shortened the time to EEG acquisition, increased physicians' confidence in diagnosis and treatment plans, and improved the accuracy of physicians' diagnoses compared to clinical judgment alone (13).

Although prior studies have examined diagnostic concordance between *Rapid-EEG* and conventional EEG montages (obtained retrospectively at single institutions), a detailed, patient-level examination of diagnostic information obtained from initial *Rapid-EEG* and subsequent conventional EEG in a multicenter prospective cohort of patients has not been previously reported. In this study, we aimed to expand on this prior work to describe seizure characteristics and associated EEG findings detected on either *Rapid-EEG* or conventional EEG, investigate whether seizures were missed on *Rapid-EEG* due to the reduction in electrode number, particularly the exclusion of midline and parasagittal coverage, and examine whether discrepancies in *Rapid-EEG* and conventional EEG findings were explained by interval events, such as changes in anti-seizure treatment.

## Methods

The DECIDE study protocol was approved by institutional review boards at participating institutions and is described in detail in the study report (13). When physicians ordered EEG monitoring for patients suspected to have non-convulsive seizures, they would then set up the *Rapid-EEG* system at the bedside (typically within minutes). Patients would be monitored with *Rapid-EEG* until the conventional EEG system could be set up by EEG technologists (typically within hours), and patients would be continuously monitored for at least 24 h with the conventional EEG system. Treatment was based on local standards-of-care, namely based on clinical suspicion or conventional EEG monitoring and not *Rapid-EEG* monitoring. Data about details of treatment after *Rapid-EEG* use were not collected in the study. Among the 164 patients whose *Rapid-EEG* data were reported in the study, we identified a subset of 22 patients who had seizures detected either on *Rapid-EEG* only (*n* = 6), conventional EEG only (*n* = 5), or both *Rapid-EEG* and conventional EEG (*n* = 11). EEG data was no longer available for 3 patients (2 with seizures on both devices, 1 with seizures only on conventional EEG), so the total number of cases included in this study was 19. This subset was clinically heterogenous except for the fact that electrographic seizures were detected in all of them. In both the original DECIDE study and this follow-up study, EEGs were visually reviewed without any automated software.

For each patient, we examined the electrographic characteristics (timing, laterality, and morphology) of seizures, especially whether seizures detected only on conventional EEG were localized to the midline or parasagittal channels. For patients who had seizures detected on only one device, we classified EEG findings on the other device either as HEP, which included abnormal epileptiform activity such as periodic discharges and lateralized rhythmic delta activity that did not meet Salzburg criteria for electrographic seizures (14), or as diffusely slow or normal background activity. We collected demographic and clinical characteristics, such as delays between *Rapid-EEG* and conventional EEG recording (approximately equal to the duration of *Rapid-EEG* monitoring since the device recorded until the conventional EEG system arrived) and prior treatment with anti-seizure medications (ASMs) or anesthetics/sedatives. Given the low number of patients in this series, statistical analysis was limited to descriptive data, including representative samples of salient electrographic patterns on conventional EEG and *Rapid-EEG*.

## Results

**Figure 1:**
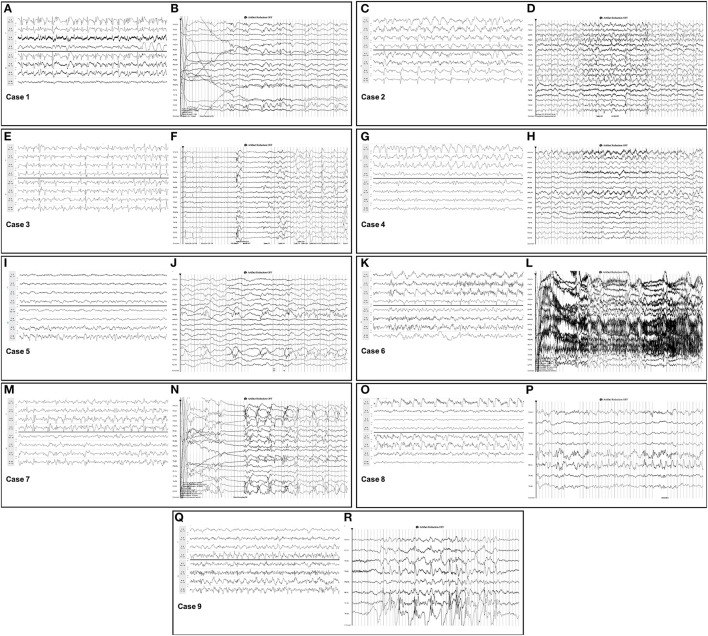
Seizures detected on *Rapid-EEG* and conventional EEG. Seizures detected on both *Rapid-EEG* (left image within each case panel) and conventional EEG (right image within each case panel) in cases 1–9 displayed similar electrographic morphology and laterality. Individual case descriptions corresponding to panels **(A–R)** are provided in Supplementary Data.

**Figure 2:**
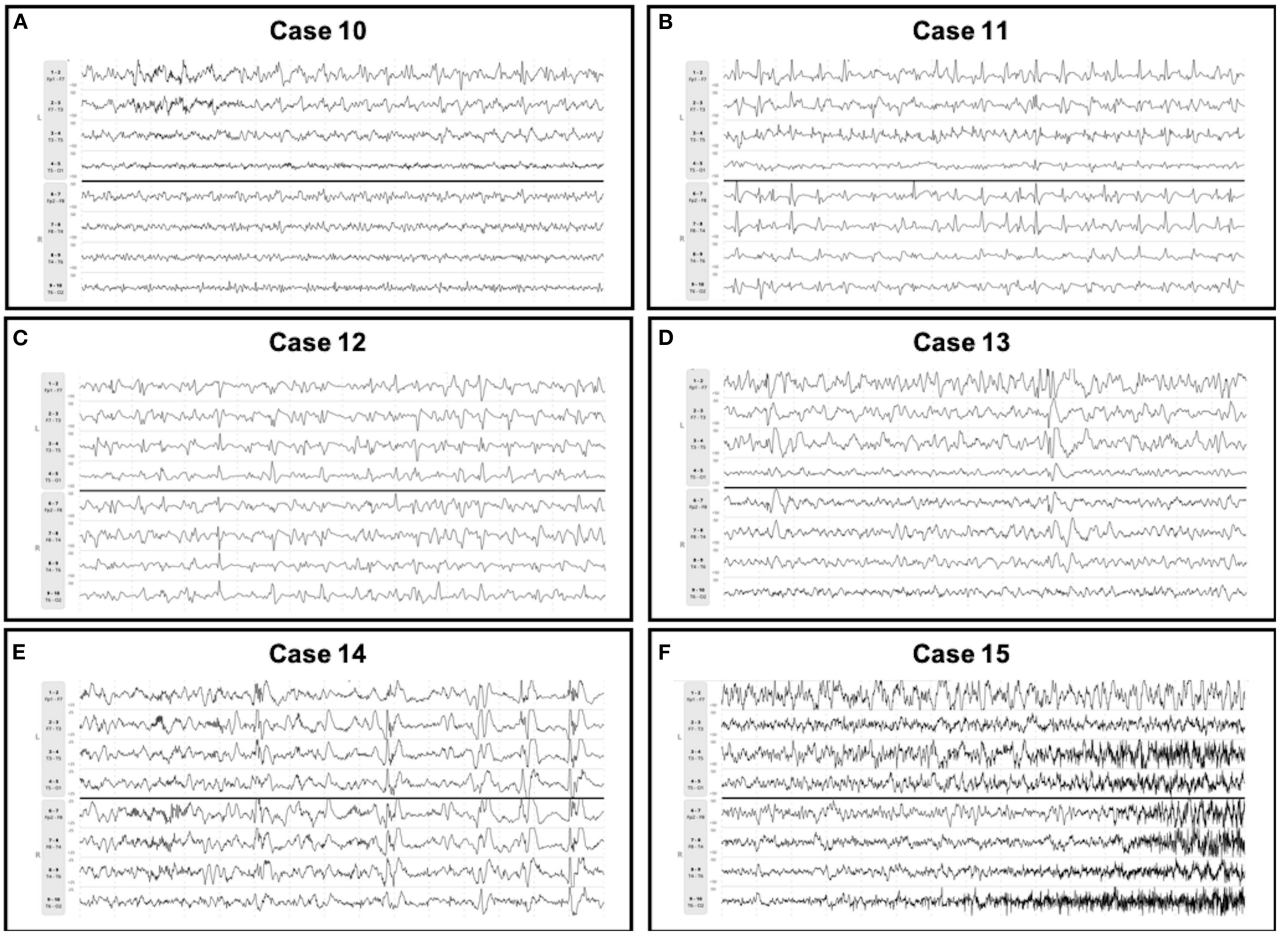
Seizures detected on *Rapid-EEG* only. Seizures detected on *Rapid-EEG* (but not conventional EEG) in cases 10–15 were appropriately treated. Subsequent conventional EEG showed HEP of similar laterality in cases 10–12 and normal activity in case 13; conventional EEG data was unavailable for cases 14 and 15. Individual case descriptions corresponding to panels **(A–F)** are provided in Supplementary Data.

**Figure 3:**
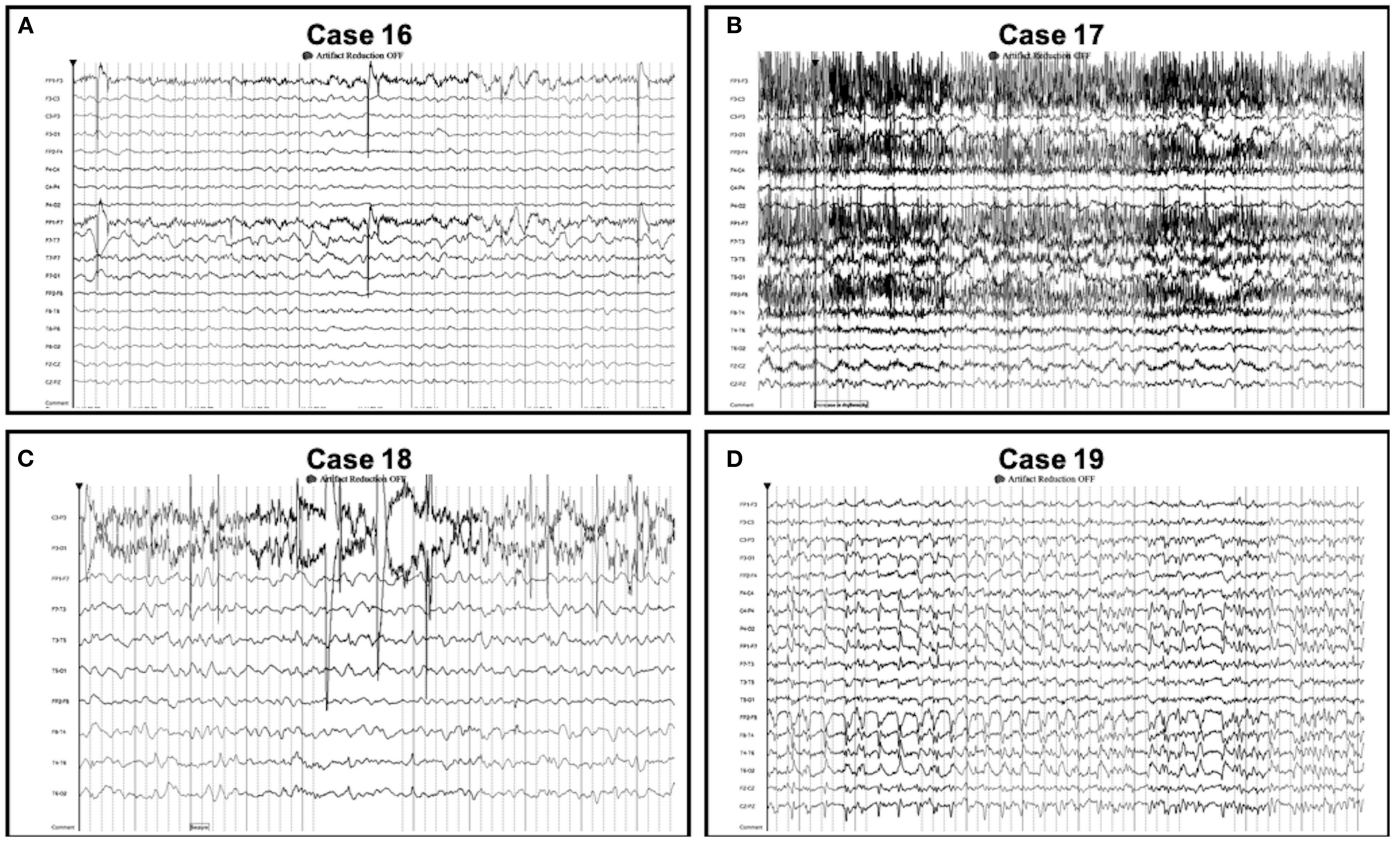
Seizures detected on conventional EEG only. Seizures detected on conventional EEG (but not *Rapid-EEG*) in cases 16–19 were not restricted to the midline or parasagittal regions. Individual case descriptions corresponding to panels **(A–D)** are provided in Supplementary Data.

Characteristics of the 19 patients with seizures detected either on *Rapid-EEG* or conventional EEG or on both devices are shown in Supplementary Table 1. EEG findings across the two EEG systems are summarized in Table 1, and representative ictal epochs from each case are shown in Figures 1–3. Case descriptions of each patient's EEG monitoring course are provided in Supplementary Data.

**Table 1:** Case series summary.

**Case**	***Rapid-EEG* diagnosis**	**Conventional EEG diagnosis**	Time to *Rapid-EEG* (in hours)*	**Time to conventional EEG (in hours)^†^**
**Seizures detected on both Rapid-EEG and conventional EEG**				
1	GPD evolving into generalized NCSE	Similar to *Rapid-EEG*	2.5	9.4
2	GPD+R evolving into generalized NCSE more prominent over left hemisphere	Similar to *Rapid-EEG*	0.8	1.5
3	Generalized NCSE maximal over bifrontal regions	Similar to *Rapid-EEG*	NA	5.5
4	Focal seizures with left frontal/temporal onset	Similar to *Rapid-EEG*	NA	1.6
5	LPD evolving into focal seizures with right parietal/occipital/temporal onset	Similar to *Rapid-EEG*	NA	2.5
6	LPD evolving into focal seizures with left hemispheric onset	Similar to *Rapid-EEG*	1.3	2.6
7	Generalized NCSE maximal over posterior quadrants	Similar to *Rapid-EEG*	NA	2.7
8	Focal seizures with right frontal onset sometimes involving left frontal region	Similar to *Rapid-EEG*	0.2	3.1
9	Focal seizures with right>left temporal/occipital onset	Similar to *Rapid-EEG*	0.2	2.0
**Seizures detected on Rapid-EEG, but not conventional EEG**				
10	BIRD evolving into focal seizures with left frontal onset	Left frontal polymorphic delta slowing and left frontal discharges, occasionally periodic at 1 Hz	0.7	16.6
11	GPD evolving into generalized NCSE	Generalized slowing and GPD at 1–2 Hz	6.5	8.7
12	Generalized NCSE	Polymorphic delta slowing and bilateral asynchronous pseudo-periodic discharges	NA	3.9
13	Focal seizures with left frontal onset	Normal activity	1.2	3.6
14	GPD evolving into brief generalized seizures	NA	NA	3.6
15	Generalized seizures	NA	0.3	NA
**Seizures detected on conventional EEG, but not Rapid-EEG**				
16	Diffuse slowing	Focal seizure with left frontal/temporal onset	2.4	2.4
17	Diffuse slowing	RDA over left > right hemispheres evolving into focal seizures maximal in left lateral and parasagittal channels	0.4	NA
18	No seizures	Seizures	0.1	8.5
19	Diffuse suppression and slowing	Generalized spikes maximal over right temporal region evolving into focal seizures	0.1	1.4

*EEG findings categorized according to American Clinical Neurophysiology Society's Standardized Critical Care EEG Terminology (15). For concordant cases, EEG diagnoses were the same between Rapid-EEG and conventional EEG so conventional EEG diagnosis is listed as “Similar to Rapid-EEG”*.

*BIRD, brief potentially ictal rhythmic discharges; GPD, generalized periodic discharges (+R indicates superimposed rhythmic activity); LPD, lateralized periodic discharges; NA, not available; NCSE, non-convulsive status epilepticus; RDA, rhythmic delta activity*.

^*^Delay to acquiring Rapid-EEG was calculated as the difference between the time at which conventional EEG was requested and the time when Rapid-EEG was set up at bedside. For some subjects, the conventional EEG request time was documented after the time Rapid-EEG monitoring started, so these were recoded as “not available”.

*†Delay to acquiring conventional EEG was approximated by the duration of Rapid-EEG monitoring, which was continued until the conventional EEG system arrived*.

For the nine concordant cases, in which both systems revealed seizures, the electrographic features of seizures and HEP across the two EEG systems had the same diagnostic and morphological qualities when reviewed by EEG experts (Figure 1). Notably, 5 of these patients had focal seizures that were captured on both EEG systems despite the lack of midline/parasagittal coverage in the *Rapid-EEG* montage; none of these seizures were restricted exclusively to the midline or parasagittal regions.

For discordant cases, clinical details of patients' hospital courses surrounding EEG monitoring (e.g., variable monitoring durations, delays in EEG acquisition, ASM treatment) seemed to affect the timing and detection of EEG patterns (Supplementary Data). We found that the 6 patients in whom seizures were detected only on *Rapid-EEG* had longer median duration of *Rapid-EEG* monitoring [3.9 h (IQR 3.6–8.7)] and greater delays in the arrival of the conventional EEG system [range: 1.5–17.3 h] compared to the 4 patients in whom seizures were detected only on conventional EEG (median *Rapid-EEG* monitoring duration: 2.4 h (IQR 1.9–5.5); range in time to conventional EEG: 1.6–8.6 h). Although these factors may explain *Rapid-EEG*'s greater yield for seizure activity in these 6 cases, we examined electrographic characteristics of seizures on *Rapid-EEG* (Figure 2) and pathological activity on subsequent conventional EEG to evaluate the possibility that *Rapid-EEG* led to false positive seizure detections. In cases 10–12, conventional EEG detected HEP of similar laterality; conventional EEG data in case 13 was read as normal after the patient had been treated with ASMs, and conventional EEG data was unavailable for cases 14 and 16. While the majority of patients were already treated with either ASMs (79%) or anesthetics/sedatives (63%), 3 of 4 patients (75%) with seizures detected only on conventional EEG were treated with ASMs or sedatives prior to *Rapid-EEG* monitoring, and later seizures detected on conventional EEG were preceded by ASM weaning. While this may explain why *Rapid-EEG* in these cases showed only diffusely slow or suppressed background activity, we also confirmed that pathological activity detected on conventional EEG (Figure 3) was not restricted to the midline or parasagittal channels absent from the *Rapid-EEG*'s hairline montage, supporting the theory that *Rapid-EEG* did not simply miss these ictal patterns due to reduced spatial coverage.

## Discussion

Our descriptive report of 19 cases of electrographic seizure from a recent multicenter prospective study (13) showed that the *Rapid-EEG* reduced montage preserved key diagnostic information compared to subsequent conventional EEG recordings using the International 10–20 system. We also found that discordance between *Rapid-EEG* and conventional EEG diagnoses was explicable when patients' clinical histories were reviewed and, at least in this case series, was not clearly attributable to the lack of midline or parasagittal coverage in *Rapid-EEG*'s reduced montage. This is the first detailed real-world case series that supports prior literature describing the diagnostic concordance between conventional and hairline EEG montages (9, 10).

In all 9 cases with seizures detected on both *Rapid-EEG* and conventional EEG systems, seizures were similar in electrographic appearance and laterality. While previous studies have described the accuracy of seizure diagnoses from brief epochs of reduced montage EEG (9, 10) and reduced electrode arrays are certainly not suited to precise seizure localization for presurgical evaluation, it is still valuable for electroencephalographers to be able to detect and describe the lateralization and evolution of seizures and their relationship to preceding or subsequent HEPs using reduced montage EEG. The 6 cases in which *Rapid-EEG* detected seizures (but conventional EEG did not) were associated with greater delays in the arrival of conventional EEG, longer durations of *Rapid-EEG* monitoring, and appropriate ASM treatment before conventional EEG monitoring. In these cases, conventional EEG often revealed HEP with the same laterality as the seizures detected on *Rapid-EEG*, arguing against the possibility that non-epileptiform patterns were over-interpreted and inappropriately treated. The four cases in which conventional EEG detected seizures (and *Rapid-EEG* did not) were confounded by several clinical factors, notably shorter *Rapid-EEG* monitoring while on ASMs/sedatives and subsequent ASM weaning during conventional EEG monitoring, and none had seizures primarily in the midline or parasagittal regions. Had *Rapid-EEG* monitoring continued longer, one could anticipate that later seizures would have been detected reliably since they were visible in the temporal chains (case 16: Fp1/F7; case 17: T3/T5; case 19: all electrodes). It is important to note that in the 164 EEG episodes included in the DECIDE trial, the cohort did not include any focal seizures strictly restricted to midline or parasagittal regions that one could argue could have been missed by *Rapid-EEG* as a result of reduced spatial coverage. However, as described elsewhere, midline and parasagittal seizures are rare in adult patients, especially critically ill populations, and when midline and parasagittal seizures occur, they are often reflected in the temporal chains (12). Future studies might prospectively assess *Rapid-EEG*'s ability to detect these focal seizures.

In detailing this clinical case series, we are aware of several limitations. These cases represent a clinically heterogenous subset of a larger clinical trial (and there were also several instances of missing electrographic data), and as such, our analysis was limited to descriptive statistics rather than significance testing. In comparing electrographic data obtained by *Rapid-EEG* and conventional EEG at different time points, we were principally interested in whether *Rapid-EEG* preserved general characteristics of ictal and interictal patterns (e.g., appearance and laterality) and whether clinical (non-EEG) factors could explain differences in seizure detection over time. We focused our description of electrographic characteristics to lateralization, rhythmicity/periodicity, and evolution, which would be of more immediate clinical value. Given the small sample size, we were not in a position to assess diagnostic accuracy due to the dynamic nature of EEG patterns (electrographic activity can be different from one time point to the next and can also be modified by changes in the patient's clinical condition) and differences in pattern classification that arise from the ability to review different montages (which is not possible with *Rapid-EEG*). Additionally, we classified EEG findings into broad groups (i.e., seizure, HEP, or non-epileptiform slow or normal activity) because it can be difficult to distinguish between specific findings (especially with less dense electrographic data). However, we would direct readers to several prior reports on the diagnostic accuracy of *Rapid-EEG*'s montage that derived the reduced EEG montage from the conventional full montage and compared diagnostic impressions between the two montages reflecting brain activity at the same point in time (9, 10). Our study findings are not necessarily specific to the *Rapid-EEG* system and could be applicable to other abbreviated EEG approaches. The use of reduced electrode arrays is seemingly at odds with the current trend of increasing electrode density for more precise spatial localization and recent recommendations to increase the number of electrodes in the standard EEG array (16), however it is important to highlight that these devices fulfill a distinct gap in clinical neurophysiology, namely emergent EEG monitoring to rule out ongoing non-convulsive status epilepticus. This need is not currently met by conventional EEG infrastructure and does not overlap with presurgical applications of high-density EEG. The opportunity to streamline conventional EEG infrastructure and facilitate rapid EEG monitoring using standard EEG arrays remains fertile ground for future investigation.

## Conclusion

In this series of patients with electrographic seizures from a multicenter prospective clinical study of *Rapid-EEG*, a recently developed reduced EEG device for rapid evaluation of suspected non-convulsive seizures and status epilepticus, the morphology and laterality of electrographic data obtained with *Rapid-EEG* were largely concordant with that obtained with subsequent conventional EEG. Clinical factors—such as variability in EEG monitoring duration, initiation or weaning of anti-seizure treatment, and changes in patients' clinical conditions—were identified as possible confounders for cases of discordant seizure detection between *Rapid-EEG* and conventional EEG.

Future studies may prospectively compare simultaneous *Rapid-EEG* and conventional EEG recordings to determine whether seizures are missed by *Rapid-EEG* due to the reduction in spatial coverage after controlling for the various patient-specific factors that affected the yield of *Rapid-EEG* and conventional EEG observed in the present study.

## Data Availability Statement

The raw data supporting the conclusions of this article will be made available by the authors, without undue reservation.

## Ethics Statement

The studies involving human participants were reviewed and approved by Institutional Review Boards at participating institutions (Massachusetts General Hospital, Rush University Medical Center, University of California Los Angeles, University of Texas Southwestern, and Wake Forest Baptist Health). The patients/participants provided their written informed consent to participate in this study. Written informed consent was obtained from the individual(s) for the publication of any potentially identifiable images or data included in this article.

## Author Contributions

JP was responsible for study conception and design. DK and KG were responsible for data acquisition, analysis, and interpretation and initial drafting of the manuscript. MD and MM contributed to data acquisition by providing EEG sample interpretations. DE, PV, and JP contributed to data interpretation. All authors contributed to the critical revision of the manuscript for intellectual content and approved of the final version for submission.

## Funding

KG received consulting fees from Ceribell Inc., for his contributions to this report. Ceribell Inc., did not participate in the collection, analysis, or interpretation of data, writing of this report, or decision to submit this report for publication.

## Conflict of Interest

JP was inventor of Rapid Response EEG System and co-founder of Ceribell Inc., which is commercializing the Rapid Response EEG System for clinical use. KG and MM serve as scientific advisors to Ceribell Inc. JP's, KG's, and MM's contributions to this manuscript were not part of their duties to Stanford University or Icahn School of Medicine at Mount Sinai. DE has received compensation for speaking or consulting from Eisai, UCB, Sunovion, Liva Nova, Neuropace, and Greenwich. PV has received travel reimbursement and compensation from Ceribell Inc., to present the results of the DECIDE clinical study. DE's and PV's institution has received funding from Ceribell Inc. The remaining authors declare that the research was conducted in the absence of any commercial or financial relationships that could be construed as a potential conflict of interest.

## Publisher's Note

All claims expressed in this article are solely those of the authors and do not necessarily represent those of their affiliated organizations, or those of the publisher, the editors and the reviewers. Any product that may be evaluated in this article, or claim that may be made by its manufacturer, is not guaranteed or endorsed by the publisher.

## Abbreviations

ASM: anti-seizure medication
BIRD: brief potentially ictal rhythmic discharge
DECIDE: Does Use of Rapid Response EEG Impact Clinical Decision Making
EEG: electroencephalography
GPD: generalized periodic discharges
HEP: highly epileptiform patterns
LPD: lateralized periodic discharges
NCS: non-convulsive seizure
NCSE: non-convulsive status epilepticus
*Rapid-EEG*: rapid response EEG
RDA: rhythmic delta activity.
