# Supplementary material for: Comparing Seizures Captured by Rapid Response EEG and Conventional EEG Recordings in a Multicenter Clinical Study
Source: Front Neurol. 2022 Jun 29;13:915385. doi: 10.3389/fneur.2022.915385 (PMC9277057; doi:10.3389/fneur.2022.915385)
Supplement: Supplementary file 1 [file Data_Sheet_1.PDF]

Supplementary Material

Supplementary Table 1. Patient characteristics

Characteristic	All patients	Seizure on both *Rapid-EEG* and conventional EEG	Seizure on *Rapid-EEG* only	Seizure on conventional EEG only
Number of patients	19	9	6	4
Age, in years (median [IQR])	51.5 [36.0-66.5]	48.0 [36.0-82.0]	35.5 [30.0-54.8]	51.5 [48.5-56.8]
Female gender, n (%)	6 (31.5)	4 (44.4)	1 (16.7)	1 (25.0)
Site, n (%)				
I	3 (15.8)	1 (11.1)	2 (33.3)	0 (0.0)
II	7 (36.8)	3 (33.3)	3 (50.0)	1 (25.0)
III	4 (21.1)	3 (33.3)	0 (0.0)	1 (25.0)
IV	3 (15.8)	2 (22.2)	0 (0.0)	1 (25.0)
V	2 (10.5)	0 (0.0)	1 (16.7)	1 (25.0)
Already treated with ASM, n (%)	15 (78.9)	7 (77.8)	5 (83.3)	3 (75.0)
Already treated with anesthetics or sedatives	12 (63.2)	5 (55.6)	4 (66.7)	3 (75.0)
Time from admission to start of *Rapid-EEG* recording, in hours (median [IQR])	6.4 [3.1-29.3]	4.0 [1.8-18.0]*	11.7 [10.0-14.3]	135.4 [4.8-269.1]†
Time from EEG order to start of *Rapid-EEG* recording, in hours (median [IQR])	0.4 [0.2-1.3]	0.5 [0.2-1.2]	0.7 [0.3-1.2]	0.3 [0.1-0.9]
Duration of *Rapid-EEG* recording, in hours (median [IQR])	3.1 [2.4-5.5]	2.6 [2.0-3.1]	3.9 [3.6-8.7]	2.4 [1.9-5.5]
Time from EEG order to start of conventional EEG recording, in hours (median [IQR])	3.3 [2.2-4.8]	2.5 [2.2-3.3]	4.8 [3.6-15.1]	4.8 [3.2-6.7]

ASM, anti-seizure medications.

*In Case 5, EEG was ordered 786.1 hours after hospital admission.

†In Cases 16 and 18, EEG was ordered 282.6 hours and 264.6 hours, respectively, after hospital admission.

Supplementary Data. Electroclinical descriptions of seizures on *Rapid-EEG* and conventional EEG

1 Seizures detected on both *Rapid-EEG* and conventional EEG (n=9)

1.1 Case 1

Both *Rapid-EEG* and conventional EEG detected generalized non-convulsive status epilepticus with a similar signal characteristic (generalized sharp waves maximal in bifrontal regions with evolution in frequency and amplitude). However, *Rapid-EEG* (**Figure 1A**) had recorded this for 5 hours 40 minutes before the arrival of the conventional EEG system (**Figure 1B**), which revealed seizure activity from the very beginning of the recording.

1.2 Case 2

Rapid-EEG (**Figure 1C**) showed waxing and waning generalized periodic discharges interspersed with evolving and faster rhythmic activity, consistent with non-convulsive seizures more predominantly over the left hemisphere. Conventional EEG arrived 33 minutes later and detected diffuse slowing, generalized periodic discharges, and seizures primarily involving the left hemisphere (**Figure 1D**).

1.3 Case 3

Rapid-EEG (**Figure 1E**) showed non-convulsive status epilepticus for 5 hours and 55 minutes before conventional EEG arrived and detected a similar pattern (**Figure 1F**). This pattern consisted of generalized sharp waves seen with maximal amplitude over the bifrontal regions, waxing and waning in frequency and amplitude throughout the recording.

1.4 Case 4

Rapid-EEG showed focal seizures in the left hemisphere with a broad onset in the frontal and temporal regions lasting from 30 seconds to less than 2 minutes without overlying myogenic activity, suggesting non-convulsive focal seizures (**Figure 1G**). Conventional EEG arrived after 16 minutes 58 seconds of *Rapid-EEG* monitoring and noted similar focal left hemispheric seizures seen over the frontal and temporal region (**Figure 1H**). In addition, both modalities demonstrated focal left hemispheric slowing.

1.5 Case 5

The entirety of *Rapid-EEG* monitoring (around 22 minutes) showed diffuse slowing and focal seizures in the right parieto-occipital and temporal channels that, at times, reverted to lateralized periodic discharges in the same channels (**Figure 1I**). This same pattern was later seen on conventional EEG (**Figure 1J**).

1.6 Case 6

Review of *Rapid-EEG* showed diffuse slowing and predominantly left hemispheric lateralized periodic discharges that evolved into focal seizures seen electrographically as left hemispheric sharp waves evolving in amplitude and frequency mixed with increased myogenic activity (**Figure 1K**). This lasted for the duration of the *Rapid-EEG* recording (1 hour and 20 minutes) until conventional

EEG arrived and showed similar findings with an increased amount of myogenic activity (**Figure 1L**).

1.7 Case 7

Continuous seizure activity with polyspike-and-wave morphology maximal in the left posterior hemisphere was seen on *Rapid-EEG* (**Figure 1M**) for around 1 hour and 46 minutes before the device was disconnected. Subsequent conventional EEG also showed seizure activity that was best appreciated on the lateral (rather than parasagittal) channels (**Figure 1N**).

1.8 Case 8

Focal right frontal seizures characterized by repetitive sharp and spike wave complexes that at times also included activity in the left frontal lead were detected intermittently on *Rapid-EEG* (**Figure 1O**) for around 3 hours before the device was disconnected and conventional EEG showed identical seizures in the right frontal leads (**Figure 1P**). Note that this site used a reduced montage of the conventional EEG system in the intensive care unit.

1.9 Case 9

Repetitive sharp and spike wave complexes over the temporo-occipital region (right more than left) evolved in frequency and amplitude into focal seizures that were seen on *Rapid-EEG* (**Figure 1Q**) for 1 hour and 45 minutes before the device was exchanged for the site's reduced conventional EEG montage, which showed similar seizure activity (**Figure 1R**).

2 Seizures detected on *Rapid-EEG*, but conventional EEG showed no seizure (n=4) or information was missing (n=2)

2.1 Case 10

EEG was requested around midnight on a Friday to evaluate encephalopathy and speech difficulties. *Rapid-EEG* monitoring took place for 27 minutes before the patient was sent for imaging, about an hour later, the patient was reconnected to *Rapid-EEG* to record for another 3 hours. During these recordings, brief ictal rhythmic discharges in the form of repetitive spike/polyspike-and-wave discharges were noted in the left frontal region, and at times, these clearly evolved and lasting longer than 10 seconds consistent with focal seizures (**Figure 2A**). The patient was treated with levetiracetam, and conventional EEG obtained about 12 hours later showed mild diffuse theta slowing, focal left frontal polymorphic delta slowing, and frequent left frontal blunt discharges occasionally periodic at 1 Hz.

2.2 Case 11

A 32-year-old man presented with cardiac arrest with return of spontaneous circulation after 14 minutes of cardiopulmonary resuscitation and subsequently underwent cooling for 24 hours at an outside hospital. In the intensive care unit, he was noted to have whole-body twitching and jerking movements, for which he was given boluses of midazolam and levetiracetam and his propofol drip was increased. EEG at the outside hospital prior to transfer reportedly showed diffuse slowing with burst suppression while on levetiracetam, phenytoin, and midazolam and propofol drips. He was transferred to the study site at midnight on a Sunday, where *Rapid-EEG* was acquired for around 8 hours and showed generalized periodic discharges with clear evolution to faster (>2-3Hz) rhythmic

activity consistent with non-convulsive status epilepticus (**Figure 2B**). Conventional EEG started around 9 hours later during business hours (at which time the patient was on valproate, levetiracetam, clobazam, and propofol and midazolam drips) and showed generalized slowing with abundant semi-rhythmic 1-2 Hz waxing and waning generalized sharp wave discharges without any associated jerking movements. However, when the propofol drip was weaned a day later, the patient developed rhythmic shaking with corresponding bifrontal myogenic artifact which continued until a bolus of propofol was given. The patient was transitioned to comfort care due to refractory status epilepticus presumed secondary to severe anoxic brain injury and passed away 2 days later.

2.3 Case 12

A 39-year-old female with drug-resistant epilepsy was admitted for suspicion of status epilepticus. *Rapid-EEG* acquired around 4am showed unequivocal non-convulsive status epilepticus for 4 hours and 35 minutes before the device was disconnected (**Figure 2C**). Patient was treated with propofol and phenobarbital in addition to her home medications (lacosamide, levetiracetam, topiramate) prior to the arrival of conventional EEG, which showed continuous polymorphic delta slowing and bilateral asynchronous pseudo-periodic discharges that resolved at the onset of the record. A marked improvement in the EEG was noted throughout the monitoring course.

2.4 Case 13

A 72-year-old man with multiple medical problems was transferred from an outside hospital for persistent and unexplained altered mental status. At the study site, *Rapid-EEG* monitoring during business hours showed repetitive spikes over the left frontal region that subsequently increased in frequency and amplitude and evolved into cyclic focal seizures in the left hemisphere during the roughly 3 hours of recording (**Figure 2D**). Subsequent conventional EEG was read as normal. Although few details about the hospital course were available, discharge records indicated that the patient was treated with valproic acid, lacosamide, and levetiracetam.

2.5 Case 14

A 30-year-old man with history of epilepsy underwent *Rapid-EEG* monitoring on a Friday evening around 8pm for evaluation of persistent encephalopathy after a witnessed generalized convulsive seizure. This recording lasted about 1 hour and 20 minutes and showed generalized epileptiform spike and wave discharges seen throughout the record that frequently evolved into brief discrete seizures with greater amplitude and frequency (**Figure 2E**). Conventional EEG information was not available for this patient.

2.6 Case 15

Around 3 hours of *Rapid-EEG* monitoring showed multiple seizures with witnessed tonic activity (**Figure 2F**). Clinical and conventional EEG information were not available for this patient.

3 Seizures detected on conventional EEG, but not *Rapid-EEG* (n=4)

3.1 Case 16

A 54-year-old man was admitted to the intensive care unit for seizures after traumatic brain injury and empirically treated with phenytoin, levetiracetam, and lacosamide. *Rapid-EEG* monitoring for 2

hours during business hours showed mild diffuse slowing without any epileptiform activity, however after *Rapid-EEG* was disconnected, levetiracetam was weaned off and the patient had a witnessed breakthrough seizure. About 12 hours into the subsequent conventional EEG, an epoch was marked as showing electrographic seizure over left frontal and temporal regions visible in lateral chains (**Figure 3A**).

3.2 Case 17

A 47-year-old female with multiple medical problems was found down after witnessed jaw clenching and poor responsiveness and was taken to an outside hospital. Concern for seizures prompted transfer to the study site for continuous EEG monitoring, and *Rapid-EEG* obtained upon arrival for 18 minutes showed only diffuse slowing. Later, conventional EEG showed runs of rhythmic delta activity becoming more apparent with higher voltage on the left than the right, maximal in both lateral (T3, T5) and parasagittal (C3, P3) electrodes, evolving into electrographic seizures (**Figure 3B**).

3.3 Case 18

A 65-year-old male with epilepsy and prior episodes of status epilepticus presented with recurrent seizures and speech disruption in the setting of an upper respiratory infection while adherent to levetiracetam and lacosamide. While in the emergency department, the patient experienced an episode of speech arrest, arm extension, and oral automatisms that lasted around 5-10 minutes and broke with lorazepam, however he remained lethargic and intermittently desaturated. *Rapid-EEG* monitoring after lorazepam administration showed no seizures, however after the patient was admitted to the intensive care unit, a subsequent reduced conventional EEG montage was read as seizures or status epilepticus, however representative snapshots of these seizures were not available (**Figure 3C**).

3.4 Case 19

A 49-year-old male was witnessed to have a generalized tonic-clonic seizure in the post-anesthesia care unit after spinal surgery and was admitted to the intensive care unit after imaging showed multifocal acute intracerebral hemorrhage. *Rapid-EEG* was obtained for 18 minutes before conventional EEG arrived, during which the patient was on a high-dose midazolam drip and Rapid-EEG showed only cardiac artifacts with severely depressed background amplitude consistent with complete sedation. Early conventional EEG monitoring also showed severe suppression and slowing of the background. After 10 hours of recording with interval weaning of sedation, frequent high-amplitude spikes were noted that evolved in amplitude and frequency, progressing to generalized spike-and-wave activity with a right hemispheric predominance (maximal amplitude over the right temporal chains) consistent with electrographic seizures (**Figure 3D**).
